# Asymmetric Hysteresis Modeling Approach Featuring “Inertial System + Shape Function” for Magnetostrictive Actuators

**DOI:** 10.3390/ma13112585

**Published:** 2020-06-05

**Authors:** Zhi-Yuan Si, Xian-Xu ‘Frank’ Bai, Li-Jun Qian

**Affiliations:** 1 Laboratory for Adaptive Structures and Intelligent Systems (LASIS), Department of Vehicle Engineering, Hefei University of Technology, Hefei 230009, China; 2016010061@mail.hfut.edu.cn (Z.-Y.S.); hfutqlj@163.com (L.-J.Q.); 2 College of Mechanical Engineering, Anhui Science and Technology University, Fengyang 233100, China

**Keywords:** magnetostriction, hysteresis, Duhem model, inertial system, shape function

## Abstract

Hysteresis of the actuators based on magnetostrictive materials influences the control performance of the application systems. It is of importance and significance to establish an effective hysteresis model for the magnetostrictive actuators for precision engineering. In this paper, based on the analysis of the Duhem model, a first-order inertial system with hysteresis characteristic under harmonic input is used to describe the hysteresis caused by the inertia of the magnetic domains of magnetostrictive materials. Shape function is employed to describe the pinning of domain walls, the interactions of different magnetic domains of magnetostrictive materials, and the saturation properties of the hysteresis. Specifically, under an architecture of “inertial system + shape function” (ISSF-Duhem model), firstly a new hysteresis model is proposed for magnetostrictive actuators. The formulation of the inertial system is constructed based on its general expression, which is capable of describing the hysteresis characteristics of magnetostrictive actuators. Then, the developed models with a Grompertz function-based shape function, a modified hyperbolic tangent function-based shape function employing an exponential function as an offset function, a one-sided dead-zone operator-based shape function are compared with each other, and further compared with the classic modified Prandtl–Ishlinskii model with a one-sided dead-zone operator. Sequentially, feasibility and capability of the proposed hysteresis model are verified and evaluated by describing and predicting the hysteresis characteristics of a commercial magnetostrictive actuator.

## 1. Introduction

Magnetostrictive materials, a smart material, produce mechanical stress and strain when exposed to external magnetic fields [1]. Because of high energy density, large output strain/force, and ultrafast response (within several microseconds), magnetostrictive materials have been widely developed/studied in various applications, such as active vibration control [2] and ultrasonic positioning [3]. However, similar to other smart material-based actuators [4,5,6,7,8], such as piezoelectric ceramic-based actuators and shape memory alloy-based actuators, the magnetostrictive actuators behave strong hysteresis nonlinearity [9,10]. Such hysteresis nonlinearity would decrease the actuating accuracy and degrade control system performance.

**Figure 1:**
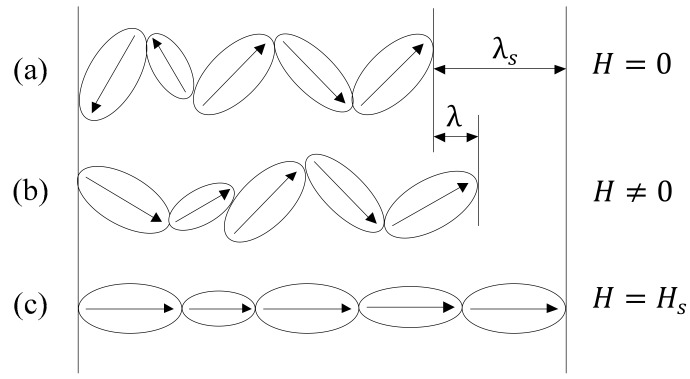
Schematic of the physical mechanism of magnetostriction. (**a**) *H* = 0; (**b**) *H* ≠ 0 (**c**) H = *H_s_*.

Physical mechanism of the magnetostriction is shown in Figure 1 (where H, $H_{s}$, λ, and $\lambda_{s}$ are the magnetic field, saturation magnetic field, magnetostriction strain, and saturation magnetostriction strain, respectively). The magnetic domains of magnetostrictive materials generate motion when exposed to external magnetic fields, which involves the motion of the domain walls and the rotation of the magnetic domains [11]. Firstly, impedances to domain wall motion due to pinning sites encountered by the domain walls as they move are the reasons for the magnetostrictive hysteresis. Secondly, the magnetic domain itself has inertia. During the motion, the magnetic domain is inevitably affected by its own inertia. Inertia would be one reason for the magnetostrictive hysteresis. Because of the non-uniformity of magnetostrictive materials, the effect of inertia on different magnetic domains is also different with each other. Thirdly, during the motion, interactions between different magnetic domains of magnetostrictive materials occur, which also appear as hysteresis macroscopically. It can also be seen from Figure 1c that the magnetostrictive material has a maximal magnetostrictive strain. This can be used to explain the saturation characteristics of magnetostrictive hysteresis.

Investigations on the nonlinearity of magnetostrictive actuators and hysteresis models have been conducted for years. Jiles–Atherton (J–A) model [11] and its extended models [12,13,14,15,16,17] were proposed to capture the hysteresis nonlinearity via the domain wall theory. However, the J-A model shows disadvantages in describing minor loops [16]. The extended J–A models are actually mainly aiming at solving such issue. In reference [18], a modified Jiles–Atherton–Sablik model is constructed for asymmetry in magnetomechanical effect under tensile and compressive stress. Operator-based hysteresis models in the phenomenological hysteresis models describe the nonlinearity by weighted superposition of elementary hysteresis operators with simple mathematical forms. The typical operator-based hysteresis models are the relay operator of the Preisach model [10,19,20,21,22], the KP kernel of the Krasnoselskii–Pokrovskii model [7,23], and the play/stop operator of the Prandtl–Ishlinskii model [24,25,26,27,28]. The disadvantages of the operator-based hysteresis models are the characteristics of the model, including symmetry and saturation, depend on the elementary hysteresis operator. The more complex the shape is, the more the number of operators will be used. In reference [29], the modified Prandtl–Ishlinskii model is constructed with a one-sided dead-zone operator (PD-Model) and used to model complex hysteresis. Another kind of phenomenological hysteresis model—differential equation-based hysteresis models—need only one auxiliary nonlinear differential equation to describe the hysteretic behavior, which shows the advantage of higher efficiency [30]. In reference [31], the Bouc–Wen model was used to describe the hysteresis nonlinearity of magnetostrictive actuators. In references [32,33], a new Duhem model is established by incorporating a shape function to describe the hysteresis of magnetorheological fluid actuators. The Duhem model describing the hysteresis characteristics of ferromagnetically soft materials was given in reference [34] but has not been found for the hysteresis characteristics of magnetostrictive materials.

The Duhem model uses a differential equation to characterize the output change with the input direction [35]. The specific input–output relationship is

(1) $$\frac{dw}{dt}=f_{1}\left(u\left(t\right),w\left(t\right)\right){\dot{u}}_{+}\left(t\right)-f_{2}\left(u\left(t\right),w\left(t\right)\right){\dot{u}}_{-}\left(t\right)$$

(2) $${\dot{u}}_{\pm }\left(t\right)=\frac{\left|\dot{u}\left(t\right)\right|\pm \dot{u}\left(t\right)}{2}$$

where $f_{1}\left(u\left(t\right),w\left(t\right)\right)$ and $f_{2}\left(u\left(t\right),w\left(t\right)\right)\in C^{0}\left(R^{2}\right)$; $w\left(t\right)$ and $u\left(t\right)$, the output and input functions, are continuous differentiable functions, respectively. When the input function $u\left(t\right)$ increases, the output function $w\left(t\right)$ increases along a path, and when $u\left(t\right)$ decreases, $w\left(t\right)$ decreases along another path.

Construction of the functions $f_{1}\left(u\left(t\right),w\left(t\right)\right)$ and $f_{2}\left(u\left(t\right),w\left(t\right)\right)$ is important for describing the system hysteresis when employing the Duhem model. The Duhem model has defined the properties of the functions $f_{1}\left(u\left(t\right),w\left(t\right)\right)$ and $f_{2}\left(u\left(t\right),w\left(t\right)\right)$, but it is still challenging to construct a suitable function based on hysteresis characteristics in practice.

Based on the analysis and literature reviewed above, especially the analysis of the physical mechanism of the hysteresis of magnetostrictive actuators, the technical contribution of this paper is that a hysteresis modeling methodology based on a first-order inertial system and shape function (ISSF-Duhem model) is constructed for magnetostrictive actuators with properties of asymmetry and saturation hysteresis. Specifically, a differential equation of the inertial system is used to represent the hysteresis caused by the inertia in the motion of magnetic domains, and a general expression of the inertial system describing the hysteresis is established. As compared with the original Duhem model, the constructed expression reduces the number of unknown functions. For the hysteresis caused by the pinning of domain walls, the interactions between the magnetic domains and the saturation characteristics of the hysteresis, a shape function is incorporated to correct the hysteresis curve represented by the inertial system. The hysteresis model will be verified and evaluated based on the description and prediction of the hysteresis of a commercial magnetostrictive actuator through experimental tests. In this study, the obtained simulation results using an ISSF-Duhem model with different shape functions (Grompertz function-based shape function, modified hyperbolic tangent function-based shape function, and one-sided dead-zone operator-based shape function) will be compared with the results obtained by using a PD-model.

## 2. ISSF-Duhem Model

According to Equation (1), the Duhem model essentially represents the hysteresis as the input-output relationship of a first-order system. Based on this, this study starts from the analysis of the hysteresis phenomenon of the inertial system and then constructs a new hysteresis model.

### 2.1. Hysteresis in an Inertial System

**Figure 2:**
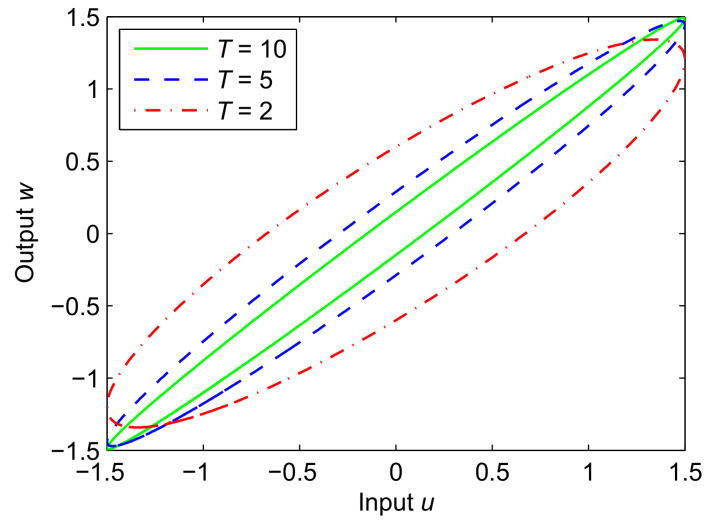
The hysteresis characteristics of the inertial system with input: *u* = 1.5sin(2π*t*), when *T* = 2, 5 and 10.

The first-order inertial system can be expressed as

(3) $$\frac{\dot{w}}{T}+w=u$$

where u, w, and T are the input, output, and the system parameter, respectively. According to Equation (3), Figure 2 presents the hysteresis performance of the inertial system. As seen from the figure, the hysteresis is related to the system parameter T.

### 2.2. Construction of Inertial System

As shown in Figure 1, the magnetic domains of magnetostrictive materials generate motion when exposed to external magnetic fields. The effect of inertia on different magnetic domains is also different with each other because of the non-uniformity of magnetostrictive materials. If the hysteresis system is considered to be a first-order inertial system, then the parameter *T* of the system is variable.

Based on reference [36], let

(4) $$T=\alpha g\left(\dot{u}\right)$$

where $\dot{u}$ is the input rate of the system; $g\left(\dot{u}\right)$ represents the function of the input rate, which is positively homogeneous; α is a constant related to the characteristics of the system.

Substituting Equation (4) into Equation (3), and to adapt to different hysteresis phenomena, expression is given as

(5) $$\dot{w}+\alpha g\left(\dot{u}\right)w=\alpha \gamma g\left(\dot{u}\right)f\left(u\right)$$

where $f_{1}\left(u\left(t\right),w\left(t\right)\right)$ is a function of u and is related to the characteristics of the hysteresis itself; γ is a constant.

Let $g\left(\dot{u}\right)=\left|\dot{u}\right|$, then Equation (5) can be rewritten as

(6) $$\dot{w}+\alpha \left|\dot{u}\right|w=\alpha \gamma \left|\dot{u}\right|f\left(u\right)$$

Equation (6) represents the inertial system hysteresis model, and its input–output relationship can be further expressed as

(7) $$\dot{w}=\alpha \left|\dot{u}\right|\left(\frac{\beta }{\alpha }f\left(u\right)-w\right)$$

where β=αγ.

Equation (7) can be further rewritten as

(8) $$\left\{\begin{array}{c} \frac{dw_{U}}{du}=\beta f\left(u\right)-\alpha w_{U}\;\;\;\;\;\;\;\;\;\;\;\dot{u}>0\\ \frac{dw_{D}}{du}=-\beta f\left(u\right)+\alpha w_{D}\;\;\;\;\;\;\;\;\;\dot{u}<0\; \end{array}\right.$$

where $\;w_{U}$ represents the value on the rising curve of the hysteresis loop; $w_{D}$ represents the value on the falling curve of the hysteresis loop. Assuming that the two endpoints of the hysteresis loop are ($u_{L}$, $w_{L}$) and ($u_{R}$, $w_{R}$), the function expression of the hysteresis loop rise and fall curves obtained by Equation (8) are

(9) $$w_{U}\left(u\right)=\frac{\beta }{\alpha }f\left(u\right)+\left[w_{L}-\frac{\beta }{\alpha }f\left(u_{L}\right)\right]e^{-\alpha \left(u-u_{L}\right)}-\frac{\beta }{\alpha }e^{-\alpha u}\int_{u_{L}}^{u}f^{\prime }\left(\varepsilon \right)e^{\alpha \varepsilon }d\varepsilon \;u\in \left(u_{L,}\;u_{R}\right)$$

(10) $$w_{D}\left(u\right)=\frac{\beta }{\alpha }f\left(u\right)-\left[w_{R}-\frac{\beta }{\alpha }f\left(u_{R}\right)\right]e^{-\alpha \left(u_{R}-u\right)}-\frac{\beta }{\alpha }e^{\alpha u}\int_{u}^{u_{R}}f^{\prime }\left(\varepsilon \right)e^{-\alpha \varepsilon }d\varepsilon \;u\in \left(u_{L,}\;u_{R}\right)$$

In Equation (9), $f^{\prime }\left(\varepsilon \right)e^{\alpha \varepsilon }$ is bounded within ($u_{L}$,$\;u_{R}$), then

(11) $$\left|\left[w_{L}-\frac{\beta }{\alpha }f\left(u_{L}\right)\right]e^{-\alpha \left(u-u_{L}\right)}-\frac{\beta }{\alpha }e^{-\alpha u}\int_{u_{L}}^{u}f^{\prime }\left(\varepsilon \right)e^{\alpha \varepsilon }d\varepsilon \right|\;\leq \left[w_{L}-\frac{\beta }{\alpha }f\left(u_{L}\right)\right]+\frac{\beta }{\alpha }f^{\prime }\left(\tau \right)e^{\alpha \tau }\left(u_{R}-u_{L}\right)=\rho_{L}$$

where $\tau \in \left(u_{L},u_{R}\right)$ and is a constant; $\rho_{L}$ is the maximal value of the absolute value of the hysteretic part on the rising curve of the hysteresis loop.

Similarly, it can be obtained by Equation (10),

(12) $$\left|-\left[w_{R}-\frac{\beta }{\alpha }f\left(u_{R}\right)\right]e^{-\alpha \left(u_{R}-u\right)}-\frac{\beta }{\alpha }e^{\alpha u}\int_{u}^{u_{R}}f^{\prime }\left(\varepsilon \right)e^{-\alpha \varepsilon }d\varepsilon \right|\;\leq \left[w_{R}-\frac{\beta }{\alpha }f\left(u_{R}\right)\right]+\frac{\beta }{\alpha }f^{\prime }\left(\varepsilon \right)e^{-\alpha \varphi }\left(u_{R}-u_{L}\right)=\rho_{R}\;$$

where $\varphi \in \left(u_{L},\;u_{R}\right)$ and is a constant; $\rho_{R}$ is the maximal value of the absolute value of the hysteretic part on the falling curve of the hysteresis loop.

Rewrite Equations (11) and (12) as

(13) $$w\left(u\right)=\frac{\beta }{\alpha }f\left(u\right)+\sigma \left(u\right)$$

(14) $$\sigma \left(u\right)=\left\{\begin{array}{c} \left[w_{L}-\frac{\beta }{\alpha }f\left(u_{L}\right)\right]e^{-\alpha u}-\frac{\beta }{\alpha }e^{-\alpha u}\int_{u_{L}}^{u}f^{\prime }\left(\varepsilon \right)e^{\alpha \varepsilon }d\varepsilon \\ -\left[w_{R}-\frac{\beta }{\alpha }f\left(u_{R}\right)\right]e^{-\alpha \left(u_{R}-u\right)}-\frac{\beta }{\alpha }e^{\alpha u}\int_{u}^{u_{R}}f^{\prime }\left(\varepsilon \right)e^{-\alpha \varepsilon }d\varepsilon \end{array}\right.$$

where

(15) $$\left|\sigma \left(u\right)\right|\leq \rho =\max\rho_{L},\rho_{R}$$

In Equation (7), $f\left(u\right)$ is a monotone increasing, piecewise smooth, odd function with a bounded derivative, that is, $\underset{u\to \infty }{\lim}f^{\prime }\left(u\right)$ is bounded. According to these conditions, the following function is selected

(16) $$f\left(u\right)=u$$

Then, Equation (7) can be rewritten as

(17) $$\dot{w}=\alpha \left|\dot{u}\right|\left(\frac{\beta }{\alpha }u-w\right)$$

### 2.3. Construction of Shape Function

The hysteresis curve of the inertial system represented by Equation (17) exhibits centrosymmetry. However, the hysteresis curve of the magnetostrictive actuators has the characteristics of asymmetry and saturation, due to the pinning of domain walls, the interactions between different magnetic domains of the magnetostrictive materials during motion, and the saturation characteristics of the magnetostrictive strain. A shape function is necessary to be incorporated to correct the hysteresis curve represented by the inertial system. In general, sigmoid functions and one-sided dead-zone operator are often used to describe the hysteresis of the magnetostrictive actuators. In this study, we construct shape functions based on these functions. It is noted that the Grompertz function, the hyperbolic tangent function, and the one-sided dead-zone operator are chosen as the basic functions for constructing the shape functions as examples; other similar functions can also be used.

#### 2.3.1. Grompertz Function-Based Shape Function

Grompertz function is employed as the shape function in this study. It is expressed as

(18) $$z_{1}\left(x\right)=-1+2e^{-e^{-\mu_{1}x-\mu_{2}}}$$

where $\mu_{1}$ and $\mu_{2}$ are both constants determining the shape of the Grompertz function output. The function $z_{1}\left(x\right)$ is within the range of [−1, 1].

When x=0,

(19) $$z_{1}\left(0\right)=-1+2e^{-e^{-\mu_{2}}}$$

It is seen that $z_{1}\left(0\right)$ is dependent on the value of $\mu_{2}$. In order to describe the hysteresis of magnetostrictive actuators, Equation (18) can be given by

(20) $$z_{1}\left(x\right)=z_{1}\left(x\right)-z_{1}\left(0\right)=2e^{-e^{-\mu_{1}x-\mu_{2}}}-2e^{-e^{-\mu_{2}}}$$

Combining Equations (17) and (20) and using w in Equation (17) as the input of the shape function, considering that the hysteresis of magnetostrictive actuators generally contains a linear component, the ISSF-Duhem model with Grompertz function-based shape function (G-Model) describing the hysteresis of magnetostrictive actuators can be given by

(21) $$\left\{\begin{array}{c} \dot{w}=\alpha \left|\dot{u}\right|\left(\frac{\beta }{\alpha }u-w\right)\;\\ z_{1}\left(w\right)=2e^{-e^{-\mu_{1}w-\mu_{2}}}-2e^{-e^{-\mu_{2}}}\\ y=k_{1}u+k_{2}z_{1} \end{array}\right.$$

where y represents the displacement of magnetostrictive actuators; $k_{1}$ and $k_{2}$ are both constants.

**Figure 3:**
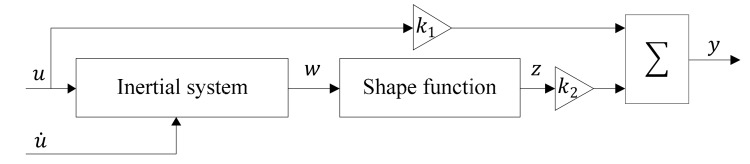
The proposed ISSF-Duhem model.

The schematic of the proposed ISSF-Duhem hysteresis model approach is presented in Figure 3. As compared with the Duhem model represented by Equation (1), the ISSF-Duhem model reduces the number of unknown functions. Only a suitable shape function is necessary to be selected according to the shape characteristics of the hysteresis curve to completely determine the model. As compared with continuous-time dynamic model of a class of backlash-like hysteresis in reference [37], formally Equation (7) of the ISSF-Duhem model is a specific case of Equation (4) of the mentioned research. However, Equation (7) has a clearer physical meaning since it is derived based on the analysis of the hysteresis of the magnetostrictive actuator, combined with characteristics of the first-order inertial system. At the same time, the hysteresis curve represented by Equation (7) is corrected by the shape function, making the ISSF-Duhem model more suitable for describing the hysteresis characteristics of magnetostrictive actuators.

#### 2.3.2. Modified Hyperbolic Tangent Function-Based Shape Function

To compare, a shape function based on the hyperbolic tangent function [38] is constructed as

(22) $$z_{2}\left(x\right)=\tanh\left(\delta x\right)$$

where δ is a constant determining the slope of the hyperbolic tangent function.

The function $z_{2}\left(x\right)$ within the range of [−1, 1] cannot be directly used as a shape function because of its centrosymmetry. A modified shape function is constructed based on the hyperbolic tangent function:

(23) $$z_{2}\left(x\right)=1+2\tanh\left(-e^{-\mu_{3}x-\mu_{4}}\right)$$

where $\mu_{3}$ and $\mu_{4}$ are both constants.

The modified hyperbolic tangent function is not centrosymmetric, which is actually constructed through offsetting the hyperbolic tangent function by exponential function $e^{-x}$. In addition, the range of modified hyperbolic tangent function is still [−1, 1] by the coefficients.

Similar to the Grompertz function, the modified hyperbolic tangent function $z_{2}\left(x\right)$ depends on $\mu_{4}$, when x=0. To describe the hysteresis of magnetostrictive actuators, Equation (23) can be rewritten as

(24) $$z_{2}\left(x\right)=2\tanh\left(-e^{-\mu_{3}x-\mu_{4}}\right)-2\tanh\left(-e^{-\mu_{4}}\right)$$

Using Equation (24) with the input of w in Equation (17) instead of $z_{1}\left(w\right)$ in Equation (21), the ISSF-Duhem model with modified hyperbolic tangent function-based shape function (M-Model) can be given by

(25) $$\left\{\begin{array}{c} \dot{w}=\alpha \left|\dot{u}\right|\left(\frac{\beta }{\alpha }u-w\right)\;\\ z_{2}\left(w\right)=2\tanh\left(-e^{-\mu_{3}w-\mu_{4}}\right)-2\tanh\left(-e^{-\mu_{4}}\right)\\ y=k_{1}u+k_{2}z_{2} \end{array}\right.$$

#### 2.3.3. One-Sided Dead-Zone Operator-Based Shape Function

To further compare, the one-sided dead-zone operator is employed as the shape function. The one-sided dead-zone operator [29] is expressed as

(26) $$\Gamma_{r_{i}}\left[x\right]=\left\{\begin{array}{c} \max\left(x-r_{i},\;0\right)\;\;\;\;\;\;\;\mathrm{for}\;\;r_{i}>0\\ \;\;\;\;\;\;\;\;\;x\;\;\;\;\;\;\;\;\;\;\;\;\;\;\;\;\;\;\;\;\;\;\;\mathrm{for}\;\;r_{i}=0\\ \min\left(x-r_{i},\;0\right)\;\;\;\;\;\;\;\mathrm{for}\;\;r_{i}<0 \end{array}\right.$$

where $r_{i}$ represents the threshold of the one-sided dead-zone operator; $\Gamma_{r_{i}}$ represents the output of the one-sided dead-zone operator with the threshold $r_{i}$.

Using Equation (26) with the input of w in Equation (17) instead of $z_{1}\left(w\right)$ in Equation (21), the ISSF-Duhem model with a one-sided dead-zone operator-based shape function (D-Model) can be given by

(27) $$\left\{\begin{array}{c} \dot{w}=\alpha \left|\dot{u}\right|\left(\frac{\beta }{\alpha }u-w\right)\;\\ z_{3}\left(w\right)=\sum_{i=-n}^{n}g_{i}\Gamma_{r_{i}}\left[w\right]\\ y=k_{1}u+k_{2}z_{3} \end{array}\right.$$

where n represents the number of one-sided dead-zone operators; $g_{i}$ represents the weighting factor of the *i*-th one-sided dead-zone operator.

When $r_{i}\geq 0$, the thresholds and weighting factors of one-sided dead-zone operators are selected as

(28) $$r_{i}=\mu_{5}i\;i=0,1,\cdots ,n$$

(29) $$g_{i}=-\mu_{6}e^{-\mu_{7}i}$$

When $r_{i}<0$, the thresholds and weighting factors of one-sided dead-zone operators are selected as

(30) $$r_{i}=\mu_{8}i\;\;i=-n,\cdots ,-n+1,-1$$

(31) $$g_{i}=\mu_{9}e^{-\mu_{10}i}$$

where $\mu_{5}$, $\mu_{6}$, $\mu_{7}$, $\mu_{8}$, $\mu_{9}$, and $\mu_{10}$ are all constants.

## 3. Experimental Validation

Aiming at verifying the performance of the ISSF-Duhem model for describing and predicting the hysteresis of magnetostrictive actuators, (i) the input–output characteristics of a commercial magnetostrictive actuator under different excitations are tested; and (ii) the ISSF-Duhem model is verified and evaluated with the measured experimental data.

### 3.1. Experimental Setup and Hysteresis Loop Test

**Figure 4:**
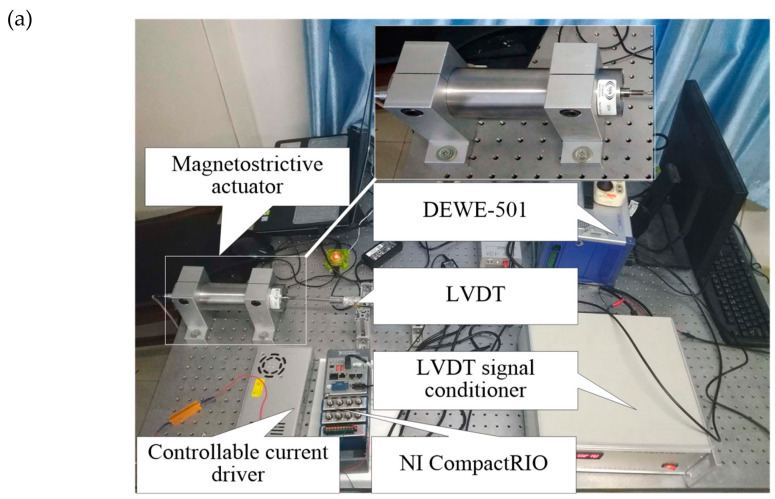

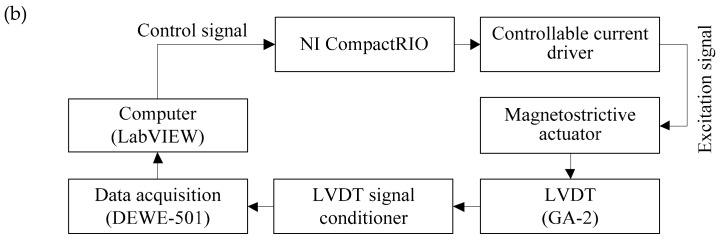
Experimental setup for characteristic tests of the magnetostrictive actuator: (**a**) the photograph and (**b**) the schematic.

Figure 4 shows the experimental setup for the hysteresis of the magnetostrictive actuator. The core component of the magnetostrictive actuator is a Terfenol-D rod with a length of 79.6 mm, a diameter of 20 mm, and the cut-off frequency of 76.07–380.34 Hz, which is prestressed about 3.023 MPa by a preload spring, and provided a bias magnetic field of about 3.2 kA/m by permanent magnets. The displacement of the magnetostrictive actuator is measured by an LVDT (Linear Variable Differential Transformer, Type: GA-2), which is a rebound type with a relatively small-stiffness spring. The excitation signal generated by LabVIEW software (version: 2014, National Instruments, Austin, TX, USA) and NI CompactRIO-9030, and amplified by a controllable current driver, is applied to the actuator. The input current and output displacement signals of the magnetostrictive actuator are acquired via the data acquisition system (Type: DEWE-501). The resonance of the mechanical setup is about 159.12 Hz.

**Figure 5:**
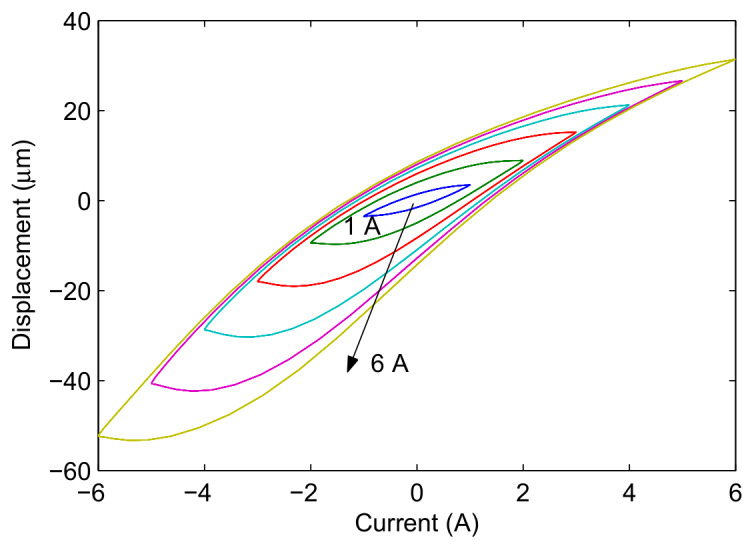
Displacement–current relationship of the magnetostrictive actuator with different excitations: *u*(*t*) = *A*_m_sin(2π*t*), *A*_m_ = 1, 2, 3, 4, 5, and 6 A.

Figure 5 presents the experimental tests in profile of the displacement versus current, when the magnetostrictive actuator is under harmonic input with different currents (1–6 A with an interval of 1 A). It can be seen that both the hysteresis and its asymmetry of the magnetostrictive actuator increases with the increase of excitation current.

**Figure 6:**
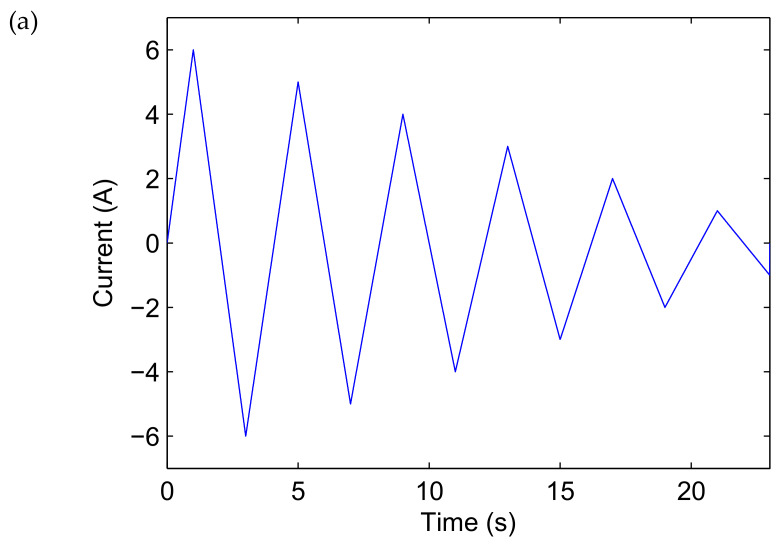

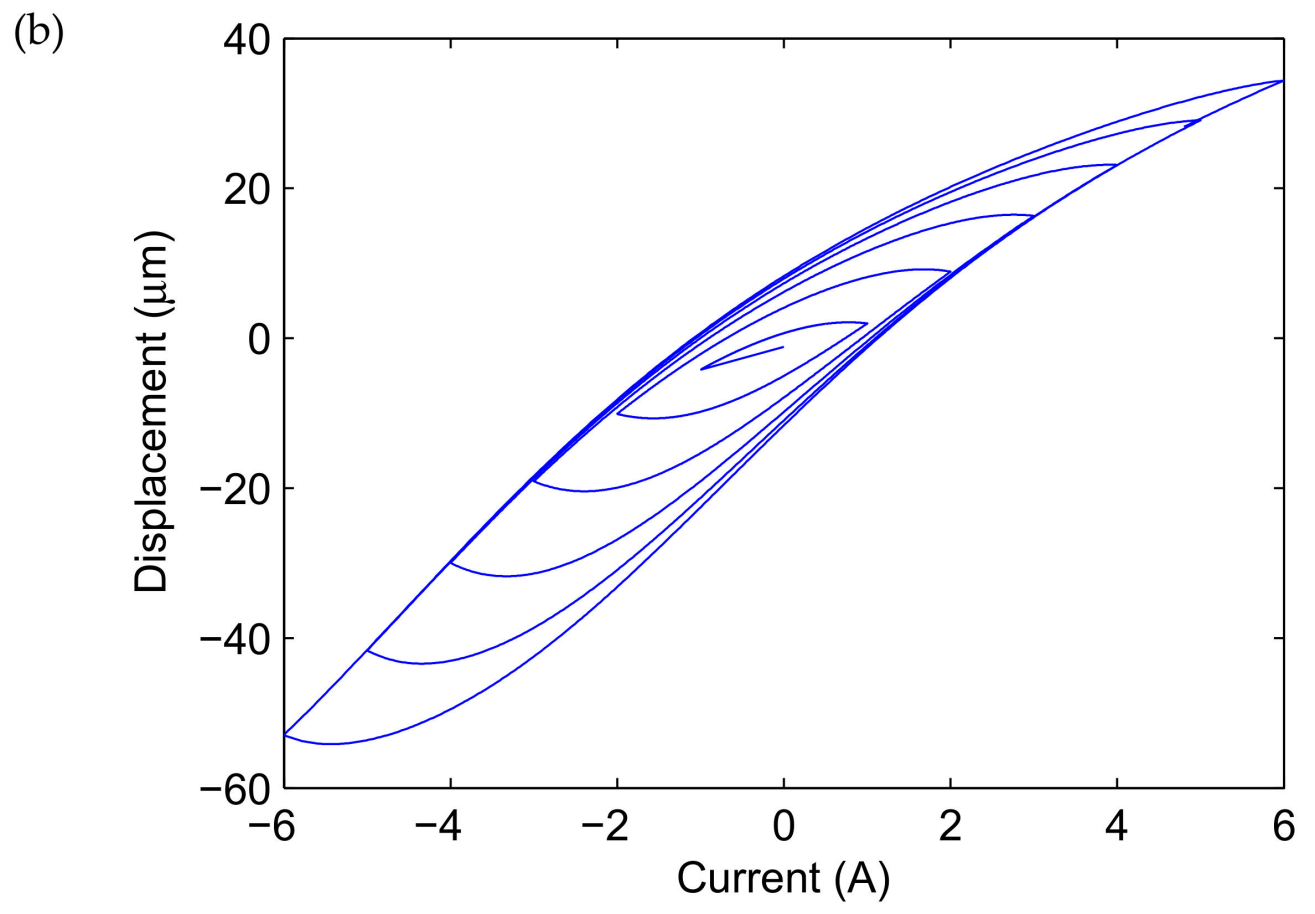
Tests of the magnetostrictive actuator under triangle excitation current: (**a**) the excitation current with different amplitudes and (**b**) the output displacement.

Figure 6a,b present the triangle excitation current with various amplitudes of 6–1 A (with a decrement of 1 A) and output displacement of the magnetostrictive actuator under the excitation current, respectively. Observing Figure 6b, both the hysteresis and its asymmetry of the magnetostrictive actuator decrease with the decrease of the current.

### 3.2. Parameter Identification

Using the experimental data of the magnetostrictive actuator under harmonic input *u*(*t*) = *A*_m_sin(2π*t*), *A*_m_ = 2, 3, 4 and 5 A, the multi-islands genetic algorithm (GA) is used to fit the established ISSF-Duhem model parameters [39]. Then, the performance of the ISSF-Duhem model is evaluated by comparing the model-based fitted results with the experimental results.

**Figure 7:**
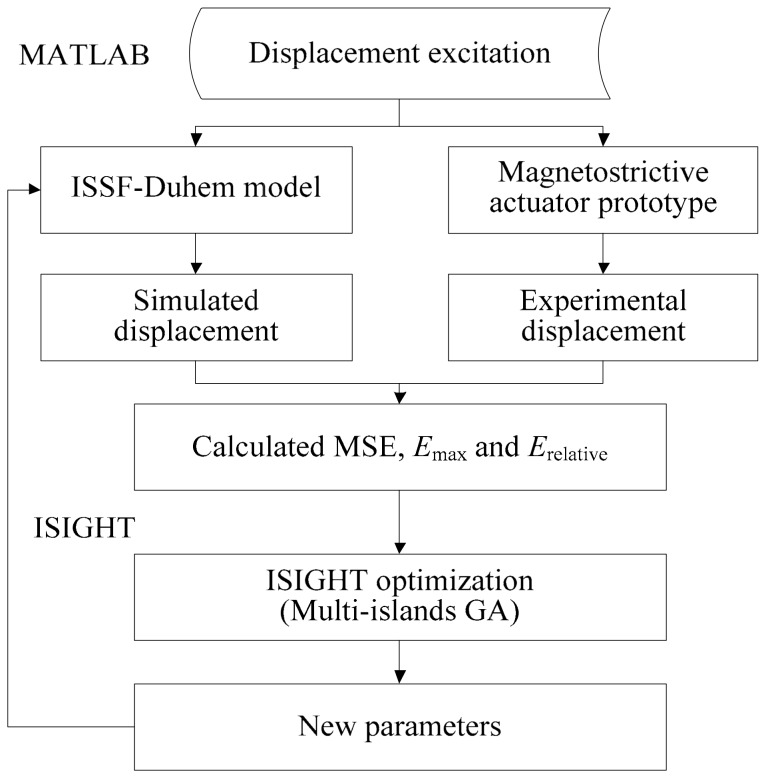
The process of parameter identification for the models.

Because of the complexity and nonlinearity of the models as represented by Equations (21), (25), and (27), and the hysteresis itself as a relatively complex nonlinear phenomenon, parameter identification becomes a key issue in this study. There are many existing methods for parameter identification, such as a least square method, modified Gauss–Newton method, differential evolution method, particle swarm algorithm, and GA. GA originates from computer simulation study on the biological evolution systems and follows the principle of natural selection and survival of the fittest. The multi-islands GA in ISIGHT software is employed for parameter identification. The specific process of parameter identification given in Figure 7 consists of two parts. One is the fitting of experimental data and the other is the update of the model parameters. The fitting process is implemented in MATLAB software, and then the fitted result is transmitted to ISIGHT software (version: 5.9.2, Dassault Systèmes Simulia Corp., Providence, RI, USA). The multi-islands GA in the ISIGHT software adjusts the values of the parameters according to the received fitted results and transmits them to the MATLAB software (version: R2014b, the Mathworks, Inc., Natick, MA, USA). Finally, the parameter identification is realized by the joint calculation of the two software programs. The results of the parameter identification are listed in Table 1. The accuracy of the hysteresis of the magnetostrictive actuator described by the ISSF-Duhem model is evaluated with the mean square error (MSE), the maximal error (*E*_max_), and relative error (*E*_relative_). MSE, *E*_max_, and *E*_relative_ are given by

(32) $$\mathrm{MSE}=\frac{1}{m}\overset{m}{\underset{i=1}{\sum }}{\left(Disp_{\mathrm{sim},i}-Disp_{\exp,i}\right)}^{2}$$

(33) $$E_{\max}=\max\left|Disp_{\mathrm{sim},\;i}-Disp_{\exp,\;i}\right|\;\;(i=1,\ldots ,m)$$

(34) $$E_{\mathrm{relative}}=\frac{E_{\max}}{\max\left(Disp_{\exp,i}\right)-\min\left(Disp_{\exp,i}\right)}$$

where *m* represents the number of data; $Disp_{\mathrm{sim},i}$ and $Disp_{\exp,\;i}$ represent *i*-th fitted and experimental displacement value, respectively; $E_{\max}$ is the maximal error; $\left|Disp_{\mathrm{sim},\;i}-Disp_{\exp,\;i}\right|$ represents the absolute value.

**Figure 8:**
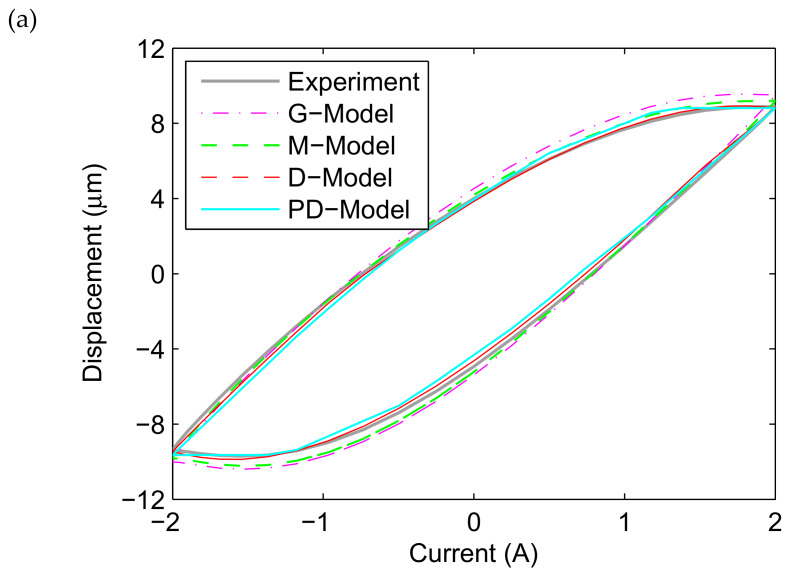

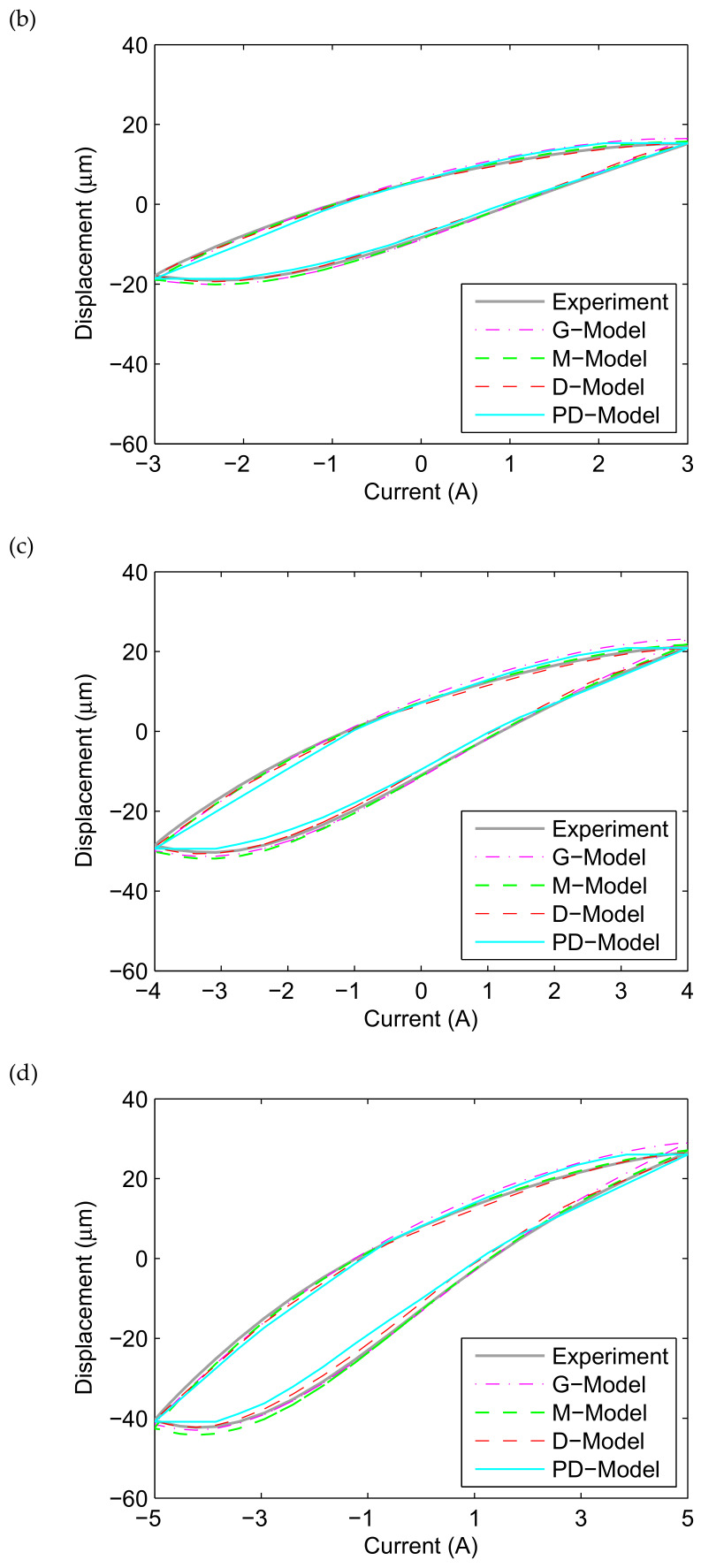
Comparison of the experimental data and the fitted results of the established ISSF-Duhem model and the PD-Model when the magnetostrictive actuator under 1 Hz harmonic input with different currents: (**a**) 2 A, (**b**) 3 A, (**c**) 4 A, and (**d**) 5 A. G-Model: the ISSF-Duhem model with Grompertz function-based shape function; M-Model: the ISSF-Duhem model with modified hyperbolic tangent function-based shape function; D-Model: the ISSF-Duhem model with a one-sided dead-zone operator-based shape function; PD-Model: the modified Prandtl–Ishlinskii model with a one-sided dead-zone operator.

Figure 8 presents the comparison between the fitted results of the ISSF-Duhem model with different shape functions as well as the PD-Model and the experimental tests in the profile of the displacement versus current, when the magnetostrictive actuator is under excitation *u*(*t*) = *A*_m_ sin(2π*t*) with *A*_m_ = 2, 3, 4, and 5 A. Observing Figure 8a, the ISSF-Duhem model can effectively describe the relationship between the displacement and current of the magnetostrictive actuator when the excitation current is 2 A. However, the fitted results of the ISSF-Duhem model with different shape functions are different. Comparing with the G-Model and M-Model, the fitted results of the D-Model are better for approximating the experimental results. At the same time, it can be seen that the fitted results of the D-Model and M-Model are better than those of the PD-Model for approximating the experimental results. Figure 8b (when *A*_m_ = 3), 8c (when *A*_m_ = 4) and 8d (when *A*_m_ = 5) show the similar results.

**Figure 9:**
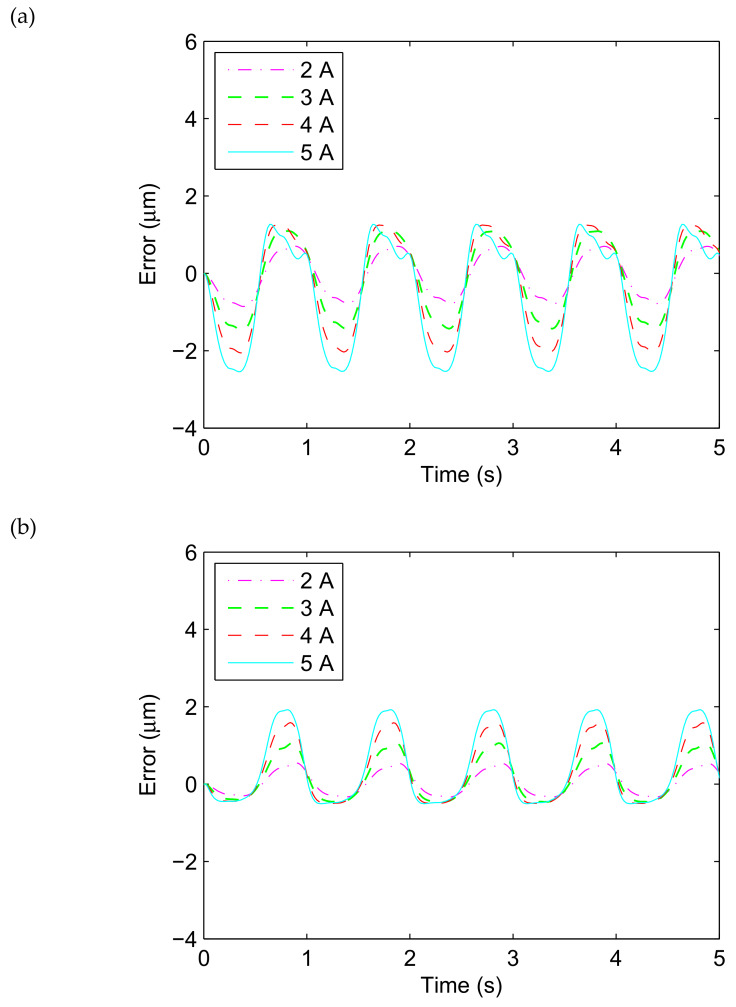

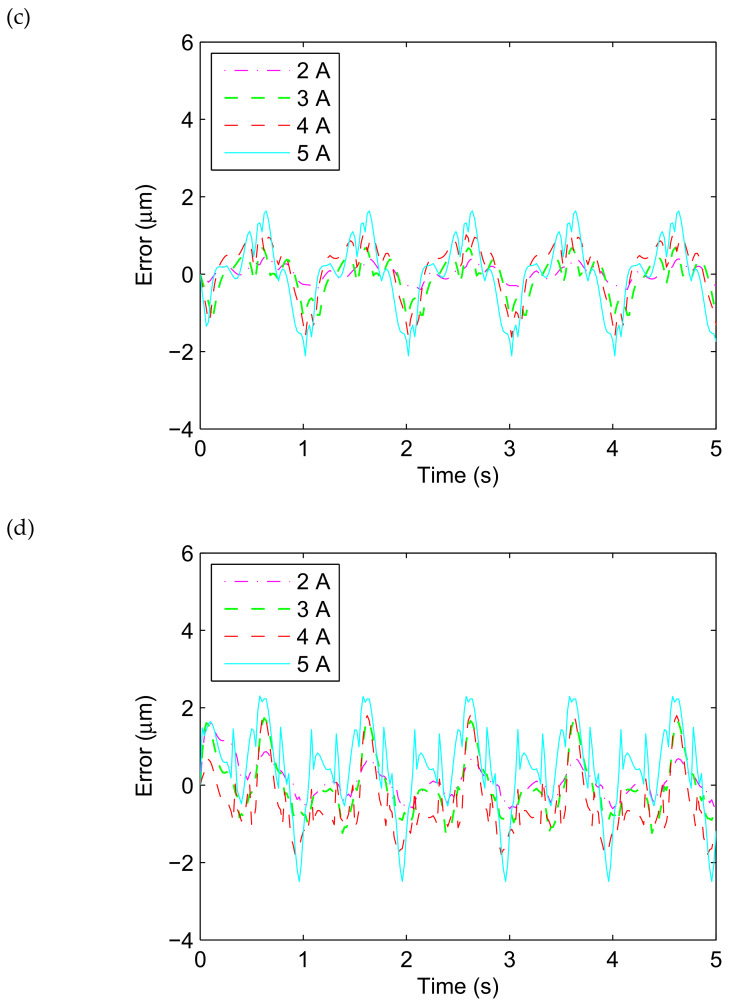
Time history of the displacement difference between experimental results and the fitted results of the established ISSF-Duhem model and the PD-Model when the magnetostrictive actuator under 1 Hz harmonic input with different currents: (**a**) G-Model: the ISSF-Duhem model with Grompertz function-based shape function, (**b**) M-Model: the ISSF-Duhem model with modified hyperbolic tangent function-based shape function, (**c**) D-Model: the ISSF-Duhem model with a one-sided dead-zone operator-based shape function, and (**d**) PD-Model: the modified Prandtl–Ishlinskii model with a one-sided dead-zone operator.

Figure 9 presents the comparison between the fitted results of the ISSF-Duhem model with different shape functions as well as the PD-Model and the experimental tests in profile of the error versus time, when the magnetostrictive actuator is under excitation *u*(*t*) = *A*_m_ sin(2π*t*) with *A*_m_ = 2, 3, 4 and 5 A. As shown in Figure 9, all the G-Model, the M-Model, the D-Model, and the PD-Model can effectively track the displacement time history of the magnetostrictive actuator and effectively describe the hysteresis with less than 3 μm error. In other words, the ISSF-Duhem model can describe the hysteresis of magnetostrictive actuators as well as the PD-Model.

**Figure 10:**
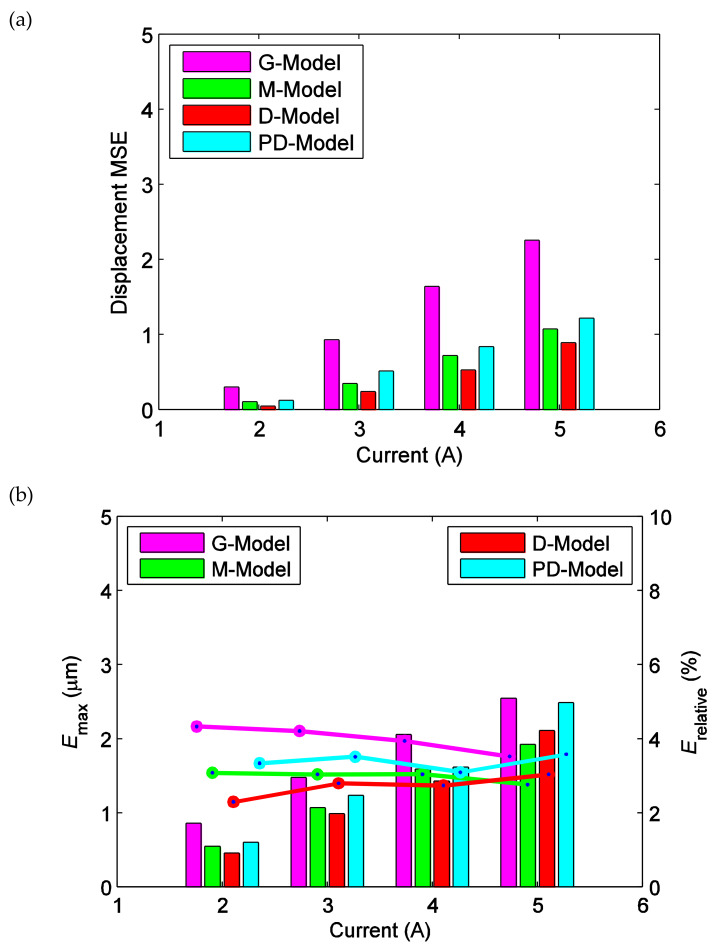
Displacement difference between experimental data and the fitted results of the established ISSF-Duhem model and the PD-Model when the magnetostrictive actuator under 1 Hz harmonic input with different currents: (**a**) MSE versus current; (**b**) *E*_max_ and *E*_relative_ versus current. *E*_max_: bars, *E*_relative_: lines. G-Model: the ISSF-Duhem model with Grompertz function-based shape function; M-Model: the ISSF-Duhem model with modified hyperbolic tangent function-based shape function; D-Model: the ISSF-Duhem model with a one-sided dead-zone operator-based shape function; PD-Model: the modified Prandtl–Ishlinskii model with a one-sided dead-zone operator.

In order to analyze the effectiveness of the ISSF-Duhem model more in depth, the fitted results of the ISSF-Duhem model and the PD-Model are further analyzed with respect to experimental results. MSE, *E*_max_, and *E*_relative_ between the fitted and experimental displacement under different excitations are calculated respectively, as shown in Figure 10. MSE reflects the overall condition of the fitted results of the ISSF-Duhem model and the PD-Model with respect to the experimental results. As seen from Figure 10a, MSE gradually increases with the increase of excitation current. When the excitation current is 5 A, MSE with a relatively small value is the largest, indicating that the ISSF-Duhem model is quite fair for the overall condition of the simulation of the hysteresis of the magnetostrictive actuator as well as the PD-Model. As shown in Figure 10a, MSE of the D-Model is smaller than that of the G-Model and M-Model. It can be concluded that, as compared with the Grompertz function-based and the modified hyperbolic tangent function-based shape function, the one-sided dead-zone operator-based shape function can enhance the performance of the ISSF-Duhem model. As seen from Figure 10a, the ISSF-Duhem model with the modified hyperbolic tangent function-based and the one-sided dead-zone operator-based shape function show better performance than the PD-Model. *E*_max_ and *E*_relative_ reflect the local condition of the fitted results of the ISSF-Duhem model and the PD-Model with respect to experimental results. Observing Figure 10b, with the increase of excitation current, *E*_max_ gradually increases, but *E*_relative_ decreases for the G-Model and the M-Model and increases for the D-Model slightly. In Figure 10b, *E*_max_ and *E*_relative_ of the D-Model are smaller than those of the G-Model, the M-Model, and the PD-model when the excitation current is less than 5 A. It indicates that the one-sided dead-zone operator-based shape function would enhance the performance of the ISSF-Duhem model. When the excitation current is 5A, *E*_max_ and *E*_relative_ of the M-Model is the smallest among the four models. Observing Figure 10a,b, the ISSF-Duhem model with the modified hyperbolic tangent function-based and the one-sided dead-zone operator-based shape function provide superior performance over the PD-Model.

### 3.3. Prediction Performance

Based on the identified parameters of the ISSF-Duhem model and the PD-Model in Section 3.2, the ISSF-Duhem model and the PD-Model are used to predict the hysteresis of the magnetostrictive actuator under two excitations: (i) *u*(*t*) = *A*_m_sin(2π*t*), *A*_m_ = 1 and 6 A; (ii) triangle excitation current with various amplitudes of 6–1 A (with a decrement of 1 A). Then, the performance of the ISSF-Duhem model is further evaluated by comparing the model-based predicted results with those of the PD-Model and with the experimental results. 

**Figure 11:**
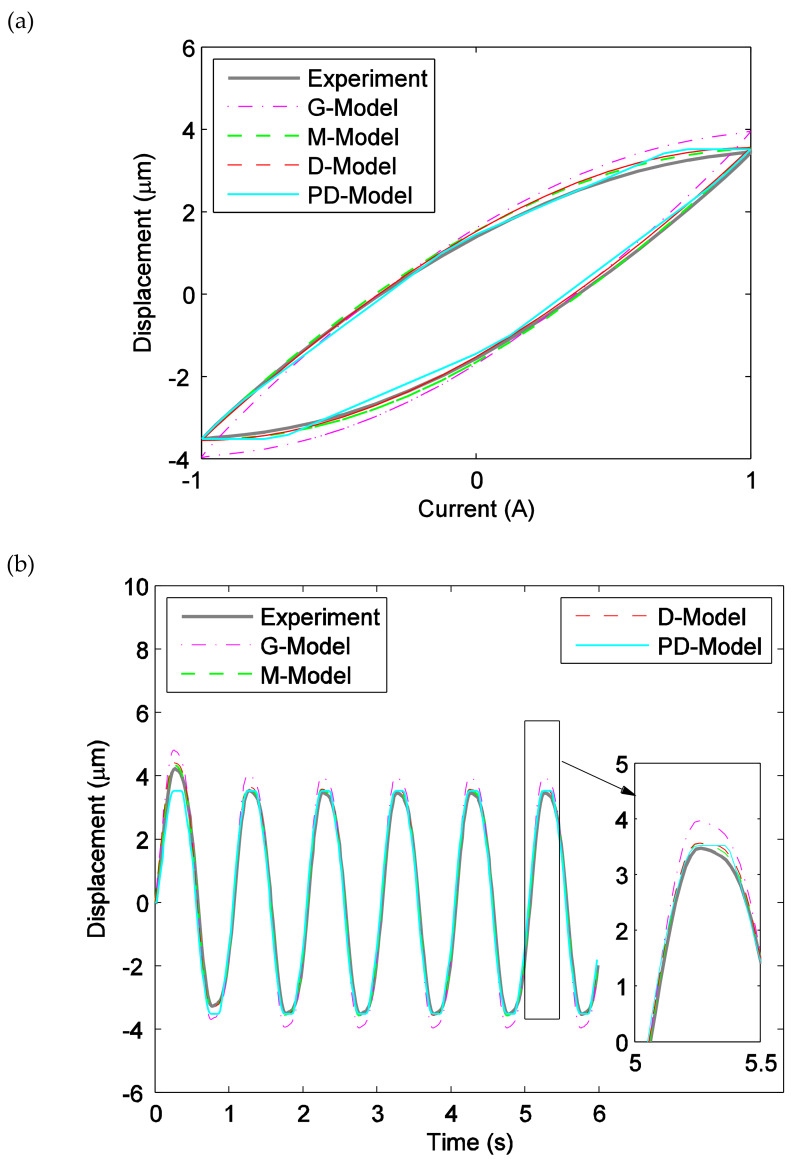

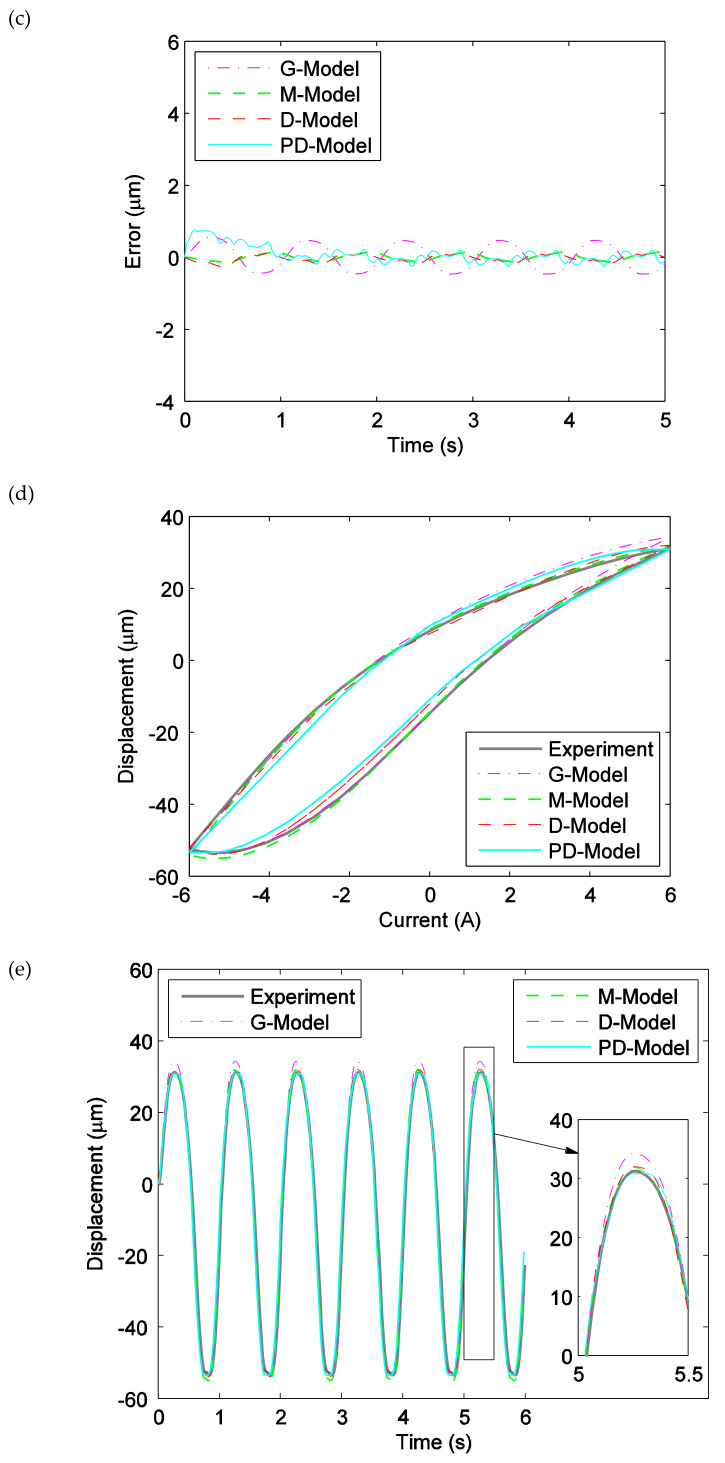

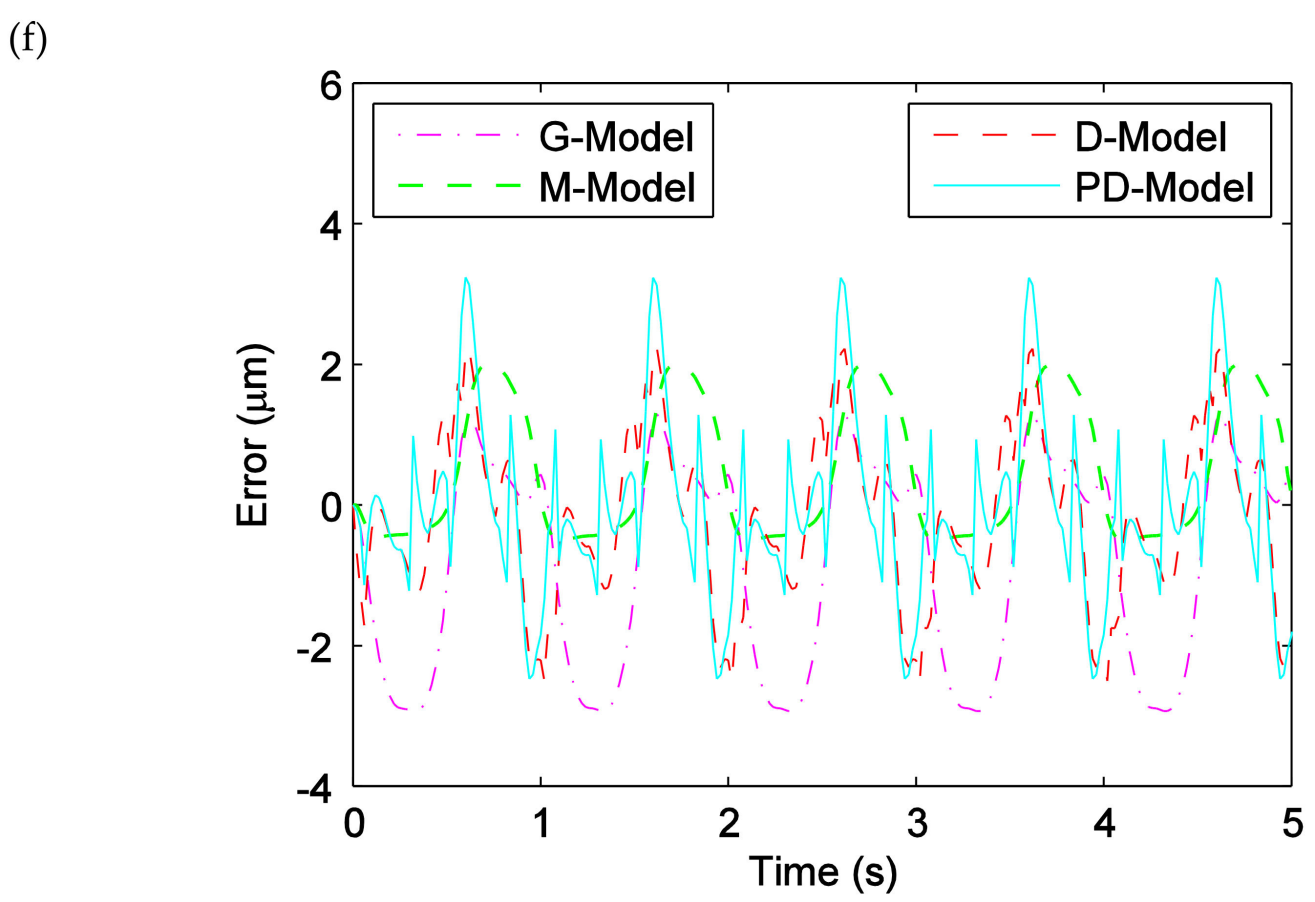
Comparison of the experimental data and the predicted results of the established ISSF-Duhem model and the PD-Model when the magnetostrictive actuator under 1 Hz harmonic input with different currents: (**a**) the displacement versus current with the current of 1 A, (**b**) the displacement versus time with the current of 1 A, (**c**) the error versus time with the current of 1 A, (**d**) the displacement versus current with the current of 6 A, (**e**) the displacement versus time with the current of 6 A, and (**f**) the error versus time with the current of 6 A. G-Model: the ISSF-Duhem model with Grompertz function-based shape function; M-Model: the ISSF-Duhem model with modified hyperbolic tangent function-based shape function; D-Model: the ISSF-Duhem model with a one-sided dead-zone operator-based shape function; PD-Model: the modified Prandtl–Ishlinskii model with a one-sided dead-zone operator.

Figure 11a–f present the comparison between the predicted results of the ISSF-Duhem model with different shape functions as well as the PD-Model with the experimental tests in profiles of the displacement versus current, the displacement versus time and the error versus time, respectively, when the magnetostrictive actuator is under excitation *u*(*t*) = *A*_m_sin(2π*t*) with *A*_m_ = 1 A and 6 A. As shown in Figure 11a–e, the ISSF-Duhem model can effectively predict the hysteresis of the magnetostrictive actuator when the excitation currents are 1 A and 6 A as well as the PD-Model. As shown in Figure 11c, when the excitation current is 1 A, the prediction errors of the M-Model, the D-Model, and the PD-Model are similar, and all smaller than the G-Model. However, when the excitation current increases to 6 A, the prediction error of the PD-Model is larger than that of the M-Model and the D-Model, as shown in Figure 11f.

**Figure 12:**
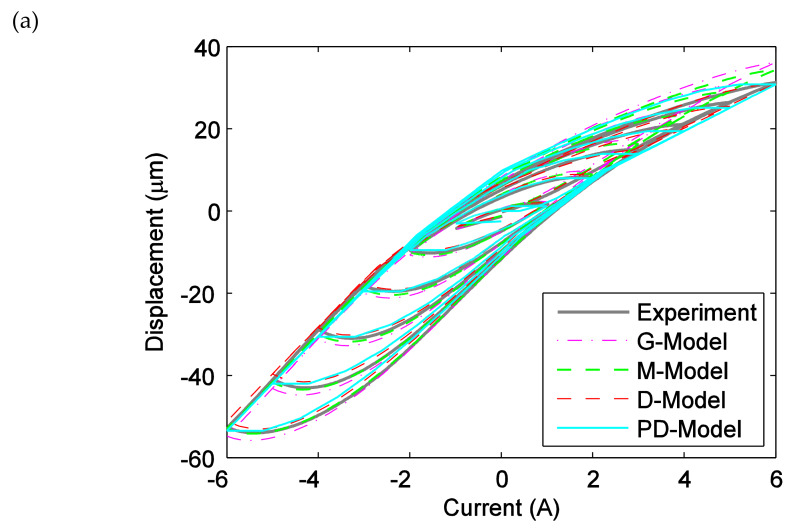

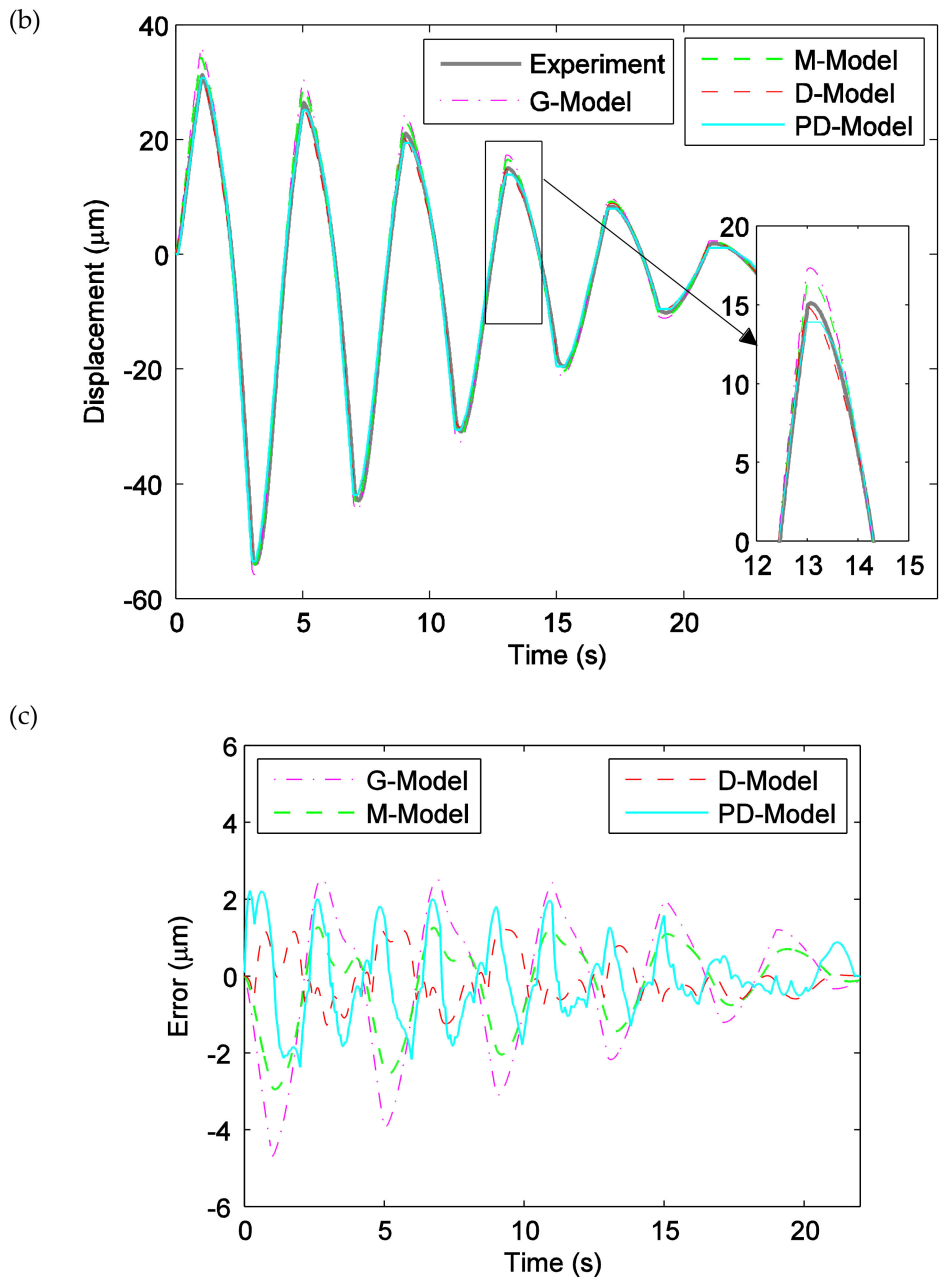
Comparison of experimental data and the predicted results of the established ISSF-Duhem model and the PD-Model when the magnetostrictive actuator under the triangle excitation current with various amplitudes: (**a**) the displacement versus current, (**b**) the displacement versus time, and (**c**) the error versus time. G-Model: the ISSF-Duhem model with Grompertz function-based shape function; M-Model: the ISSF-Duhem model with modified hyperbolic tangent function-based shape function; D-Model: the ISSF-Duhem model with a one-sided dead-zone operator-based shape function; PD-Model: the modified Prandtl–Ishlinskii model with a one-sided dead-zone operator.

Figure 12a–c presents the comparison between the predicted results of the ISSF-Duhem model with different shape functions as well as the PD-Model and the experimental tests in profiles of the displacement versus current, the displacement versus time, and the error versus time, respectively, when the magnetostrictive actuator is under the triangle excitation current with various amplitudes. Observing Figure 12, the ISSF-Duhem model can effectively predict the hysteresis of the magnetostrictive actuator under the triangle excitation and the PD-Model.

For the predicted results shown in Figure 11 and Figure 12, MSE, *E*_max_, and *E*_relative_ between the predicted and the experimental displacements are calculated, as listed in Table 2. It is seen that the ISSF-Duhem model with different shape functions has different prediction accuracy for the hysteresis of the magnetostrictive actuator under different excitations. When the harmonic excitation current is 1 A, MSE of the D-Model of the predicted result is smaller than those of predicted results of the G-Model, M-Model, and PD-Model. *E*_max_ and *E*_relative_ of the predicted results of the D-Model, the M-Model, and the PD-Model are almost the same, but all lower than that of the G-Model. When the harmonic excitation current is 6 A, MSE of the D-Model predicted result is also smaller than those of the predicted results of the G-Model, the M-Model, and the PD-Model, but *E*_max_ and *E*_relative_ of the M-Model predicted result are the smallest. When the excitation current is altered to the triangle form as shown in Figure 6a, MSE, *E*_max_, and *E*_relative_ of the D-Model predicted results are smaller than those of predicted results of the G-Model, the M-Model, and the PD-Model.

**Figure 13:**
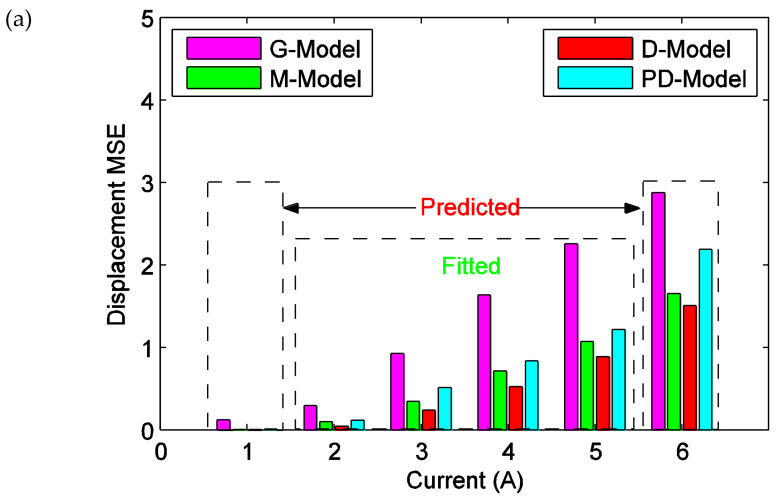

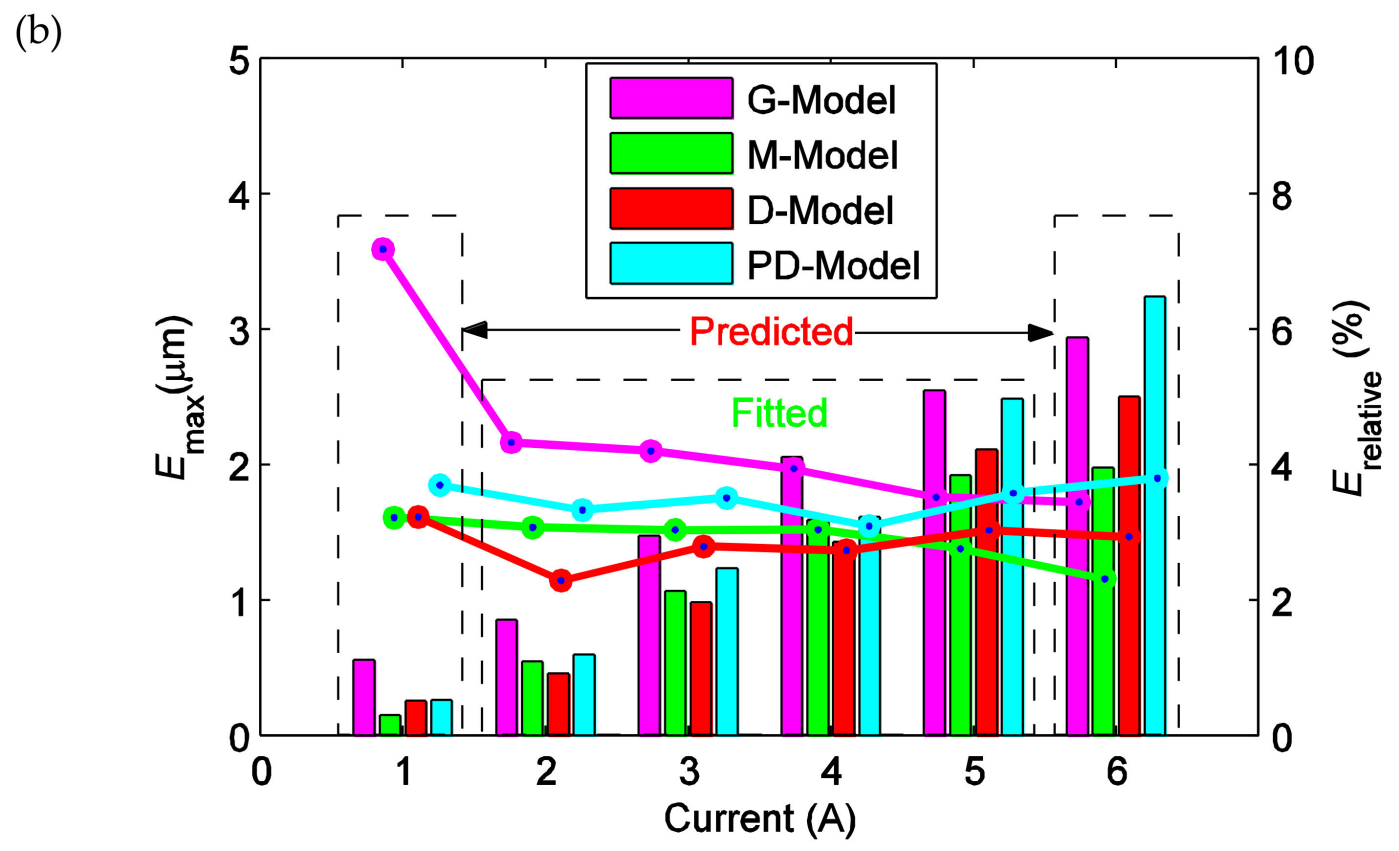
Displacement difference between the experimental results and the fitted, predicted results of the established ISSF-Duhem model and the PD-Model when the magnetostrictive actuator under 1 Hz harmonic input with different currents: (**a**) MSE versus current and (**b**) *E*_max_ and *E*_relative_ versus current. *E*_max_: bars, *E*_relative_: lines. G-Model: the ISSF-Duhem model with Grompertz function-based shape function; M-Model: the ISSF-Duhem model with modified hyperbolic tangent function-based shape function; D-Model: the ISSF-Duhem model with a one-sided dead-zone operator-based shape function; PD-Model: the modified Prandtl–Ishlinskii model with a one-sided dead-zone operator.

To sum up, MSE, *E*_max_, and *E*_relative_ of the fitted and predicted results are comprehensively analyzed and shown in Figure 13. It can be seen from Figure 13a that MSE of the predicted result of the excitation current of 6 A is significantly larger than that of the fitted result of the excitation current of 5 A. The main reason is that the hysteresis increases with the excitation current increasing. As seen from Figure 13, the one-sided dead-zone operator-based shape function can enhance the performance of the ISSF-Duhem model as compared to the Grompertz function-based and the modified hyperbolic tangent function-based shape function. However, as shown in Figure 13b, the one-sided dead-zone operator-based shape function cannot make the performance of the ISSF-Duhem model as good as the modified hyperbolic tangent function-based shape function in some local condition. Based on the above comparison and analysis, the proposed modeling approach using the inertial system combining any shape function would be helpful for modeling asymmetric hysteresis of smart material-based actuators.

## 4. Discussion

The established ISSF-Duhem model focuses only on the asymmetric hysteresis of magnetostrictive actuators and the effect of stress and temperature on their hysteresis characteristics are not considered in this study. In fact, modeling the hysteresis with consideration of the effect of stress and temperature on hysteresis itself is an interesting and challenging task. It will be investigated in the future on the basis of this work.

As regarding the inverse problems of the nonlinear hysteresis models, it is actually to establish the mathematical relationship between the target output $y_{desire}$ and the required input u ($=f_{3}\left(y_{\mathrm{desire}}\right)$). In order to inverse the ISSF-Duhem, firstly, the explicit functional relationship between w and u should be established according to the general expression of the first-order inertial system, $w=f_{4}\left(u\right)$. Secondly, substituting the explicit functional relationship between w and u into the shape function to get the function relationship between $z_{i}\left(i=1,2\right)$ and u, $z_{i}\left(i=1,\;2\right)=f_{5}\left(u\right)$. Lastly, the mathematical relationship between the target output $y_{\mathrm{desire}}$ and the required input u, i.e., the inverse model of the ISSF-Duhem model is established. The general expression of the first-order inertial system shown as Equation (7) is a differential equation, which requires a discrete relationship in order to establish the functional relationship between w and u [40]. However, due to the coupling term between w and u in the ISSF-Duhem model, the explicit function relationship between w and u cannot be obtained, so the inverse model of the ISSF-Duhem model cannot be obtained. Big data and artificial intelligence methods might be used to describe nonlinear phenomena and to obtain the inverse model of the ISSF-Duhem model [41,42,43].

## 5. Conclusions

Based on the analyses of the Duhem model and the causes of hysteresis of magnetostrictive materials, introducing the concept of inertial system and shape function, the ISSF-Duhem model for describing the hysteresis of magnetostrictive actuators featured with saturation and asymmetry is proposed and investigated in this paper. Specifically, the hysteresis caused by the inertia in the motion of magnetic domains is described by a differential equation of the inertial system. The hysteresis curve represented by the inertial system is corrected by incorporation of a shape function for the hysteresis caused by the pinning of domain walls, the interactions between the magnetic domains and the saturation characteristics of the hysteresis.

The feasibility of the ISSF-Duhem model for describing the hysteresis is analyzed by solving the model. A multi-islands GA is applied for parameter identification for the ISSF-Duhem model. According to the identified parameters, the hysteresis of the magnetostrictive actuator is simulated and predicted by the ISSF-Duhem model. The comparison between the model simulated and predicted results and the experimental results show that the ISSF-Duhem model can effectively characterize the hysteresis of magnetostrictive actuators. 

The shape function affects the performance of the ISSF-Duhem model. The comparative analysis shows that compared with the Grompertz function-based and the modified hyperbolic tangent function-based shape function, the one-sided dead-zone operator-based shape function could enhance the performance of the ISSF-Duhem model. In addition, the ISSF-Duhem model with the one-sided dead-zone operator-based and the modified hyperbolic tangent function-based shape function provide superior performance over the modified Prandtl–Ishlinskii model with a one-sided dead-zone operator.

Precise and simple hysteresis models of actuators are the foundation to realize the accurate, rapid, and effective control systems. The concept of the developed approach of the ISSF-Duhem model is of help and significance for hysteresis modeling of the magnetostrictive material-based actuators. Compared to the conventional Duhem model, the ISSF-Duhem model and the modeling approach with incorporation of the inertial system and shape function reduce the number of unknown functions. Only a suitable shape function is necessary to be selected according to the shape characteristics of the hysteresis curve to completely determine the model. As compared with the continuous-time dynamic model of a class of backlash-like hysteresis, the proposed ISSF-Duhem model has better flexibility in describing different hysteresis phenomena, and is more suitable for describing the hysteresis of magnetostrictive actuators featured with saturation and asymmetry, since it incorporates a shape function. Therefore, the modeling approach using an inertial system combining any shape function would be helpful for modeling asymmetric hysteresis of smart material-based actuators.

## Figures and Tables

**Table 1:** Parameter identification results for ISSF-Duhem model with three shape functions under 1 Hz harmonic input with different currents. G-Model: the ISSF-Duhem model with Grompertz function-based shape function; M-Model: the ISSF-Duhem model with modified hyperbolic tangent function-based shape function; D-Model: the ISSF-Duhem model with a one-sided dead-zone operator-based shape function.

Parameters	G-Model	M-Model	D-Model
$k_{1}$	3.037	2.950	2.979
$k_{2}$	34.925	33.102	0.778
α	0.593	0.612	0.575
β	3.468	3.322	1.859
$\mu_{1}$	0.059	—	—
$\mu_{2}$	0.988	—	—
$\mu_{3}$	—	0.061	—
$\mu_{4}$	—	1.251	—
$\mu_{5}$	—	—	0.452
$\mu_{6}$	—	—	2.378
$\mu_{7}$	—	—	0.098
$\mu_{8}$	—	—	0.587
$\mu_{9}$	—	—	3.997
$\mu_{10}$	—	—	0.214
n	—	—	6

**Table 2:** MSE, *E*_max_ and *E*_relative_ of the displacement difference between the predicted results of the ISSF-Duhem model and PD-Model with the identified parameters and the experimental tests when the magnetostrictive actuator is under 1 Hz harmonic input at different currents: 1 A and 6 A, and the triangle excitation current with various amplitudes. G-Model: the ISSF-Duhem model with Grompertz function-based shape function; M-Model: the ISSF-Duhem model with modified hyperbolic tangent function-based shape function; D-Model: the ISSF-Duhem model with a one-sided dead-zone operator-based shape function; PD-Model: the modified Prandtl–Ishlinskii model with a one-sided dead-zone operator. Tri: the triangle excitation current with various amplitudes.

	Error	MSE			*E*_max_ (μm)			*E*_relative_ (%)		
Model		1 A	6 A	Tri	1 A	6 A	Tri	1 A	6 A	Tri
G-Model		0.123	2.8783	2.8227	0.5583	2.9368	4.6929	7.20	3.46	5.50
M-Model		0.0184	1.6529	1.1267	0.2504	1.9796	2.9526	3.23	2.34	3.46
D-Model		0.0097	1.5047	0.9210	0.2584	2.5032	1.2861	3.24	2.95	1.53
PD-Model		0.014	2.1883	1.4272	0.2613	3.2383	2.3738	3.72	3.82	2.82
